# Penicillin Binding Proteins and β-Lactamases of Mycobacterium tuberculosis: Reexamination of the Historical Paradigm

**DOI:** 10.1128/msphere.00039-22

**Published:** 2022-02-23

**Authors:** Gaurav Kumar, Christos Galanis, Hunter R. Batchelder, Craig A. Townsend, Gyanu Lamichhane

**Affiliations:** a Division of Infectious Diseases, Department of Medicine, School of Medicine, Johns Hopkins University School of Medicine, Baltimore, Maryland, USA; b Department of Chemistry, Johns Hopkins University, Baltimore, Maryland, USA; Antimicrobial Development Specialists, LLC

**Keywords:** *Mycobacterium tuberculosis*, penicillin binding proteins, β-lactams, β-lactamase

## Abstract

Penicillin binding proteins (PBPs) have been extensively studied due to their importance to the physiology of bacterial cell wall peptidoglycan and as targets of the most widely used class of antibiotics, the β-lactams. The existing paradigm asserts that PBPs catalyze the final step of peptidoglycan biosynthesis, and β-lactams inhibit their activities. According to this paradigm, a distinct enzyme class, β-lactamases, exists to inactivate β-lactams. This paradigm has been the basis for how bacterial diseases are treated with β-lactams. We tested whether this historical view accurately reflects the relationship between β-lactams and the PBPs and the β-lactamase, BlaC, of Mycobacterium tuberculosis. BlaC was the major inactivator of the cephalosporin subclass of β-lactams. However, the PBPs PonA1 and PonA2 inactivated penicillins and carbapenems more effectively than BlaC. These findings demonstrate that select M. tuberculosis PBPs are effective at inactivating several β-lactams. Lesser-known PBPs, DacB, DacB1, DacB2, and Rv2864c, a putative PBP, were comparably more resistant to inhibition by all β-lactam subclasses. Additionally, Rv1730c exhibited low affinity to most β-lactams. Based on these findings, we conclude that in M. tuberculosis, BlaC is not the only source of inactivation of β-lactams. Therefore, the historical paradigm does not accurately describe the relationship between β-lactams and M. tuberculosis.

**IMPORTANCE** M. tuberculosis, the causative agent of tuberculosis, kills more humans than any other bacterium. β-lactams are the most widely used class of antibiotics to treat bacterial infections. Unlike in the historical model that describes the relationship between β-lactams and M. tuberculosis, we find that M. tuberculosis penicillin binding proteins are able to inactivate select β-lactams with high efficiency.

## INTRODUCTION

Penicillin binding proteins (PBPs) are a class of enzymes that are present in virtually all bacteria (1, 2). Their native function is the synthesis of cell wall peptidoglycan (PG), the exoskeleton of the bacterial cell (3–5). PG is essential for cell shape, growth, division, and viability (6, 7). Inhibition of PG synthesis is the basis for the activity of β-lactams (8). This single class of antibiotics comprises >50% of all antibiotics used to treat bacterial infections in humans (9). Due to the clinical significance of β-lactams, extensive studies have been undertaken to determine the mechanistic basis of their activities using model organisms such as Staphylococcus aureus, Bacillus subtilis, and Escherichia coli. Since similar observations were made in these organisms, a paradigm that describes the mechanistic basis of β-lactam activity was developed and assumed to be generally applicable to a wide range of bacteria (10–14). According to this historical view, two categories of proteins exist in bacteria that are relevant to β-lactam activity. The first are the PBPs, which β-lactams bind to and inhibit. The second are the β-lactamases, enzymes that inactivate β-lactams. We asked if this paradigm accurately describes the relationship between β-lactams and Mycobacterium tuberculosis, a bacterium with an atypical PG (15, 16).

M. tuberculosis, the causative agent of tuberculosis, kills more humans than any other bacterium (17). The PG of M. tuberculosis is synthesized by two enzyme classes, the PBPs and l,d-transpeptidases (18). Several independent investigations of interactions between β-lactams and l,d-transpeptidases of M. tuberculosis have reported that carbapenems and penems preferentially inhibit this enzyme class (19–27). However, for the PBPs, interactions of only a few PBPs of M. tuberculosis and with a select few β-lactams have been described (28–34). Therefore, there is a critical gap in our understanding of how β-lactams interact with the full spectrum of PBPs in M. tuberculosis and whether the historical view can explain the relationship between β-lactams and M. tuberculosis.

There is no consensus on how many and which proteins in M. tuberculosis are PBPs, so we have included all known and putative PBPs in our study based on their homology to known PBPs in other bacteria or the presence of SXXK, KTG, and SXN motifs that are characteristic of PBPs (35). High-molecular-mass (HMM) PBPs include PonA1, PonA2, PbpA, and PbpB and putative HMM PBP Rv2864c. Low-molecular-mass (LMM) PBPs include DacB, DacB1, DacB2, and potentially, Rv0907, Rv1367, Rv1730, and Rv1922 (36). The M. tuberculosis genome encodes four proteins with β-lactamase activity—BlaC, Rv0406c, Rv3677c, and CrfA (37–40). Among these, BlaC is the most potent β-lactamase (37, 41). Therefore, we have included BlaC in this study.

There are five major subclasses of β-lactams in clinical use today; these are the penicillins, cephalosporins, monobactams, carbapenems, and penems. As M. tuberculosis encodes several distinct PBPs, we hypothesize that they display a wide range of affinities to β-lactams and that not all PBPs are susceptible to inactivation by any single β-lactam. Since PBPs and β-lactamases are evolutionarily related enzymes and use a similar catalytic mechanism, we also hypothesize that there is a possibility that some PBPs may exhibit β-lactamase activity.

To test these hypotheses, we have determined affinities and inhibitory activities of representatives of all β-lactam subclasses against all known PBPs of M. tuberculosis and their ability to inactivate β-lactams. In addition, we have also determined inhibition of M. tuberculosis PBPs by T405, a newly developed β-lactam of the penem subclass (42), in order to expand the limited knowledge on how this subclass interacts with M. tuberculosis PBPs since there is only one other penem commercially available, faropenem.

## RESULTS

### Penicillins and carbapenems are effectively inactivated by PonA1 and PonA2.

**FIG 1:**
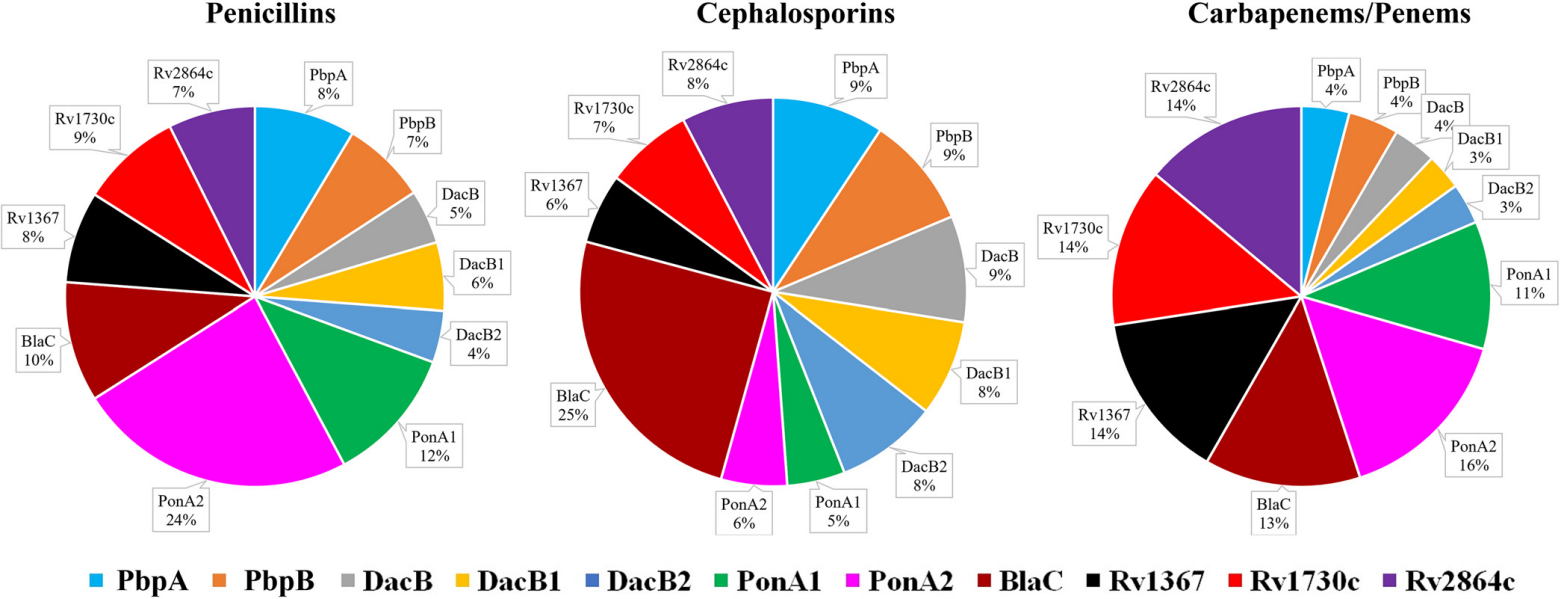
Hydrolysis of β-lactams by M. tuberculosis PBPs. The percentage hydrolysis represents the cumulative hydrolysis of each class of β-lactams by each protein. The penicillin subclasses of β-lactams included in this study are penicillin G, ampicillin, amoxicillin, piperacillin, and oxacillin. The cephalosporins included are cefadroxil, cefoxitin, cefotaxime, ceftriaxone, cefdinir, and cephalexin; the carbapenems included are imipenem, meropenem, doripenem, and biapenem, and the penems included are faropenem and T405. Additional details are included in Fig. S1.

**Inactivation of β-lactams by PBPs.** The PBPs were expressed using pET28a+TEV, which harbors kanamycin resistance marker (19) to avoid β-lactamase-based selection, as minute contamination arising from the plasmid-encoded proteins may impact β-lactamase assay. The amount of each β-lactam hydrolyzed by unit protein was measured. To determine the percent hydrolysis of each β-lactam subclass by each individual PBP, the rates at which each β-lactam was hydrolyzed by each protein were compared. Results from this experiment are summarized in Fig. 1 and Table 1 (additional information in Tables S1 and S2 and Fig. S1).

**TABLE 1:**
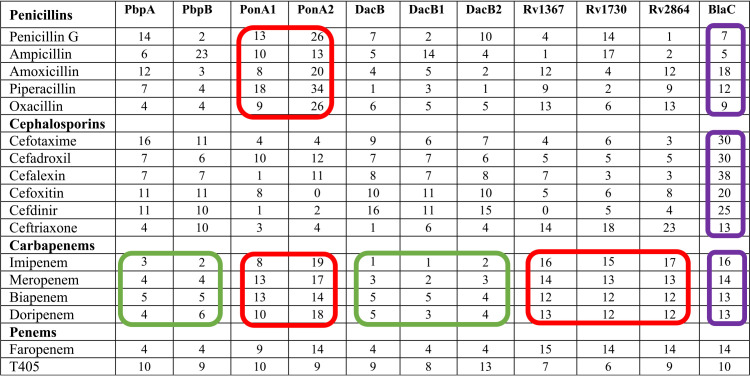
Hydrolysis (%) of individual β-lactam by M. tuberculosis PBPs^*a*^

a The amount of each β-lactam that is hydrolyzed by a PBP as a percentage of the total amount of β-lactams in each subclass hydrolyzed by all PBPs is shown. Reaction conditions such as buffer, protein and β-lactam concentrations, temperature, time course, etc. were identical for all PBP-β-lactam pairs. β-lactam subclasses that are more effectively or comparably hydrolyzed by PBPs relative to BlaC are circled in red. The subclasses that is least hydrolyzed by PbpA, PbpB, DacB, DacB1, and DacB2 are circled in green. Hydrolysis of these β-lactams by BlaC are circled in purple. Data for each PBP and β-lactam are included in Table S2.

10.1128/msphere.00039-22.1 TABLE S1 Biochemical parameters for β-lactams used in enzyme kinetic studies. Download Table S1, TIF file, 0.4 MB. Copyright © 2022 Kumar et al. 2022 Kumar et al. https://creativecommons.org/licenses/by/4.0/ This content is distributed under the terms of the Creative Commons Attribution 4.0 International license.

10.1128/msphere.00039-22.2 TABLE S2 Amount of each β-lactam hydrolyzed by M. tuberculosis unit protein expressed in nmoles (in the right column, the amount of the β-lactam hydrolyzed by each protein as a percentage of the total is displayed). Download Table S2, TIF file, 1.6 MB. Copyright © 2022 Kumar et al. 2022 Kumar et al. https://creativecommons.org/licenses/by/4.0/ This content is distributed under the terms of the Creative Commons Attribution 4.0 International license.

10.1128/msphere.00039-22.5 FIG S1 Hydrolysis of β-lactams by M. tuberculosis PBPs. The amount of each β-lactam hydrolyzed by a PBP was measured for 120 min. Reaction conditions for all PBP-β-lactam pairs were identical. Each assay was performed in triplicate under identical reaction conditions. The mean and standard error are reported. Download FIG S1, TIF file, 1.3 MB. Copyright © 2022 Kumar et al. 2022 Kumar et al. https://creativecommons.org/licenses/by/4.0/ This content is distributed under the terms of the Creative Commons Attribution 4.0 International license.

10.1128/msphere.00039-22.6 FIG S2 Hydrolysis of nitrocefin by M. tuberculosis PBPs in the presence of a β-lactam. Each panel shows nitrocefin hydrolyzed by a specific M. tuberculosis protein. Each M. tuberculosis PBP or BlaC was incubated with a β-lactam for 30 min, following which, nitrocefin was added. Hydrolysis of nitrocefin was monitored for 120 min. Each assay was performed in triplicate under identical reaction conditions. The mean and standard error are reported. Download FIG S2, TIF file, 1.1 MB. Copyright © 2022 Kumar et al. 2022 Kumar et al. https://creativecommons.org/licenses/by/4.0/ This content is distributed under the terms of the Creative Commons Attribution 4.0 International license.

PonA2 exhibited a dominant inactivation profile against penicillins, as 24% of total hydrolytic activity against this β-lactam subclass was exhibited by this protein. PonA1, with 12% of the total hydrolytic activity against penicillins, was also more potent than BlaC (10%) (Fig. 1, Table 1). Other PBPs, carboxypeptidases (DacBs), and putative PBPs exhibited less potent activity against the penicillins. PonA2 also effectively inactivated the carbapenem subclass, as 16% of the composite hydrolysis of this β-lactam subclass was attributable to this single protein. To our surprise, Rv1367c, Rv1730c, and Rv2864c, which are annotated to exhibit PBP-like function (36), and are not known to exhibit β-lactamase activities, inactivated carbapenems at a rate comparable (14% each) to that of BlaC (13%). PonA1 and BlaC also exhibited high levels of carbapenem hydrolytic activity (11% and 13%, respectively). PbpA, PbpB, and DacB were the least effective in hydrolyzing the carbapenems.

BlaC exhibited the most potent hydrolyzing activity (25%) against the cephalosporins. The levels of inactivation of cephalosporins by all other proteins were similar to each other with less than 10% each, while PonA1 and PonA2 were the least effective at inactivating this β-lactam subclass. The less-well-known putative PBPs Rv1367, Rv1730, and Rv2864 hydrolyzed ceftriaxone at a higher rate than other proteins, including BlaC (Table 1).

We extended these data to determine the overall susceptibility of each β-lactam subclass to the PBPs (Fig. 1, Table 1, Fig. S1). In general, penicillins and cephalosporins were hydrolyzed more effectively than carbapenems. Among the penicillins, penicillin G was hydrolyzed readily (>100 μM) by all proteins, followed by ampicillin and amoxicillin, whereas oxacillin and piperacillin were not hydrolyzed to the same extent. Among cephalosporins, cephalexin was the most readily hydrolyzed by BlaC (Fig. S1), while ceftriaxone was most stable against it. All proteins except PonA2 exhibited enhanced hydrolytic activity against cefoxitin (>100 nmol). Among carbapenems, imipenem was the least hydrolyzed (<50 nmol) by PBPs, followed by biapenem (<100 nmol). Moreover, doripenem and faropenem (a penem) were selectively hydrolyzed at higher levels (≥100 nmol) by PonA1, PonA2, BlaC, Rv1367c, Rv1730c, and Rv2864c, whereas, a smaller amount (∼100 nmol) of meropenem was hydrolyzed by the same proteins. Smaller amounts of carbapenems (<70 nmol) were hydrolyzed by PbpA, PbpB, DacB, DacB1, and DacB2. Overall, compared to other subclasses, carbapenems were hydrolyzed at a lower rate by the PBPs and therefore appeared to be more stable in the presence of these proteins (Fig. S1).

**Inactivation of a β-lactam reporter by M. tuberculosis PBPs.** In the above-described assay, one of the surprising findings was that PonA1 and PonA2 were more effective than BlaC at inactivating most penicillins and carbapenems. Based on these findings, we hypothesized the following: exposure of a protein to a β-lactam that it hydrolyzed at a high rate will not inhibit its β-lactamase activity. To test this hypothesis, we used on orthogonal approach based on determination of the amount of nitrocefin hydrolyzed by each PBP in the presence of each β-lactam. Nitrocefin is a commonly used chromogenic reporter for monitoring PBP hydrolytic activity (43).

**FIG 2:**
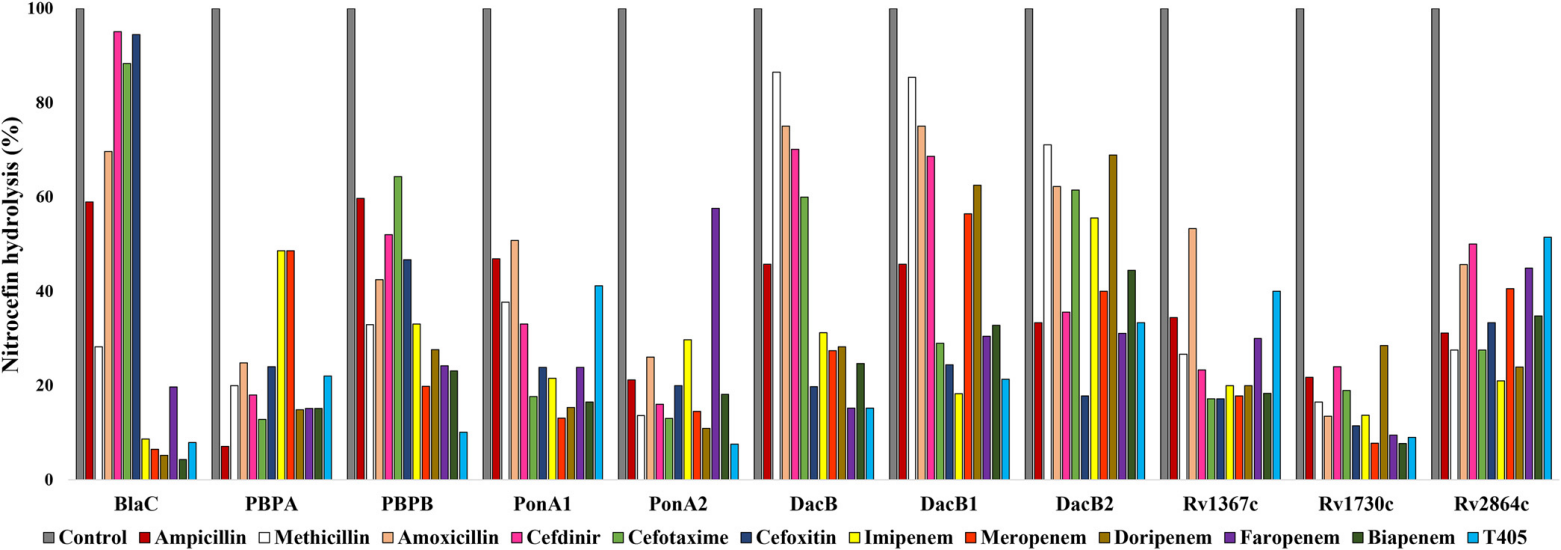
Hydrolysis of nitrocefin by M. tuberculosis PBPs in the presence of β-lactams. The percentages of nitrocefin hydrolyzed by unit PBP in the presence of unit β-lactam under identical reaction conditions are shown. Nitrocefin hydrolyzed in the absence of β-lactam is considered 100% (control), and the amounts of nitrocefin hydrolyzed in the presence of β-lactam are represented in comparison to the control. Additional details are included in Fig. S2.

Nitrocefin hydrolysis by PonA1 and PonA2 was least impaired in the presence of penicillins and carbapenems and most impaired in the presence of cephalosporins (Fig. 2, Fig. S2). In this assay as well, penicillins and carbapenems were better substrates for PonA1 and PonA2 than cephalosporins. In the β-lactam inactivation assay described above, BlaC was the dominant inactivator of cephalosporins. In this assessment, we observe that when preincubated with cephalosporins (cefdinir, cefotaxime, or cefoxitin), BlaC hydrolyzed nitrocefin at a rate comparable to the control, in which BlaC was incubated with nitrocefin alone. The rate of hydrolysis of nitrocefin by BlaC was reduced by ∼10-fold by all carbapenems compared to reductions by cephalosporins or the no-drug control. The DacB family of proteins (DacB, DacB1, and DacB2) were most effective in inactivating cephalosporins and least effective against carbapenems (Fig. 1). However, in this assay, the rates at which this protein family hydrolyzed nitrocefin when exposed to carbapenems or cephalosporins were not dissimilar.

### PbpB exhibits the highest affinity to β-lactams, whereas Rv1730 exhibits the lowest.

We undertook another orthogonal approach to generate additional insight into the binding of β-lactams by M. tuberculosis PBPs. An additional validated method to determine the binding affinity of ligands to PBPs is based on competitive binding with BOCILLIN FL, a fluorescent penicillin substrate (44). In this assay, a PBP is incubated with increasing concentrations of a β-lactam, followed by BOCILLIN FL addition. The concentration of a β-lactam that is required to inhibit BOCILLIN FL binding, and therefore reduce its fluorescence by 50% (FIC_50_), is then determined (Table 2, Table S3, Fig. S3). A high FIC_50_ indicates that the specific β-lactam is more weakly bound by the protein than a penicillin core. To begin, we screened all M. tuberculosis PBPs for their β-lactamase activity against BOCILLIN FL, as this assay requires that protein-BOCILLIN FL form a stable complex. All but BlaC, Rv1367, and Rv2864 formed stable complexes (Fig. S3). We were unable to detect any fluorescence when BOCILLIN FL was incubated with these proteins and concluded that they readily hydrolyzed and released BOCILLIN FL, as noted previously (45).

**TABLE 2:**
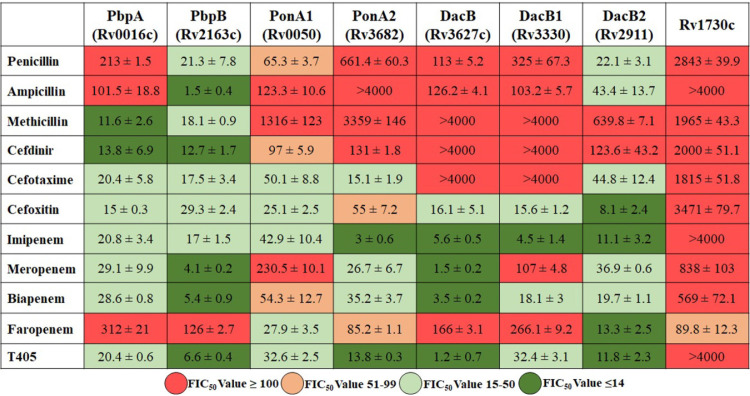
Affinity of PBPs for β-lactams^*a*^

a Fluorescence inhibitory concentrations (FIC_50_), the concentration (μM) of a β-lactam required to reduce fluorescence of BOCILLIN FL bound to a PBP to 50% of the maximum for each β-lactam and PBP pair, are shown. Additional details are included in Table S3.

10.1128/msphere.00039-22.3 TABLE S3 Biochemical parameters for BOCILLIN FL fluorescence inhibition studies. Download Table S3, TIF file, 0.2 MB. Copyright © 2022 Kumar et al. 2022 Kumar et al. https://creativecommons.org/licenses/by/4.0/ This content is distributed under the terms of the Creative Commons Attribution 4.0 International license.

10.1128/msphere.00039-22.7 FIG S3 Verification of BOCILLIN FL binding to M. tuberculosis PBPs and BlaC. Fluorescence of BOCILLIN FL (arbitrary units) with BlaC, Rv1367c, and Rv2864c could not be detected. Download FIG S3, TIF file, 0.4 MB. Copyright © 2022 Kumar et al. 2022 Kumar et al. https://creativecommons.org/licenses/by/4.0/ This content is distributed under the terms of the Creative Commons Attribution 4.0 International license.

10.1128/msphere.00039-22.4 TABLE S4 List of M. tuberculosis PBP gene locus IDs and sequences of DNA oligomers used to amplify DNA of each gene. Download Table S4, TIF file, 0.6 MB. Copyright © 2022 Kumar et al. 2022 Kumar et al. https://creativecommons.org/licenses/by/4.0/ This content is distributed under the terms of the Creative Commons Attribution 4.0 International license.

The FIC_50_ of each β-lactam and PBP pair is shown in Table 2. We will limit to general trends or observations that are not predictable from precedent (14). The FIC_50_ of most penicillins were among the highest observed. This outcome is expected as BOCILLIN belongs to the penicillin subclass, and therefore, it is unlikely that another penicillin is preferably bound by the proteins. However, an important exception was observed. The FIC_50_ of all β-lactams (except for faropenem) was consistently lowest for PbpB compared to other PBPs, indicating that this PBP has the highest binding affinity for penicillins, cephalosporins, and carbapenems. These data are in full accord with the inactivation results (Table 1) and nitrocefin hydrolysis rates described above (Fig. 2). Another surprising finding was that the FIC_50_ of all β-lactam subclasses against Rv1730c was consistently the highest, suggesting that Rv1730c binds to all β-lactam subclasses with very low affinity. In this assay, we tested two penems, faropenem and T405 (42). While T405 was bound strongly by all PBPs, binding of faropenem with all PBPs was weak except for DacB2 and PonA1. In general, the FIC_50_ of cephalosporins and carbapenems for PbpA, PbpB, PonA1, and PonA2 were low, demonstrating that these HMM PBPs bind strongly to these two β-lactam subclasses requiring low concentrations to inhibit BOCILLIN FL binding.

### Experimental penem T405 strongly binds to and inhibits M. tuberculosis PBPs.

Among the carbapenems and penems, T405 was least susceptible to inactivation by BlaC (Table 1). This observation was reproducible in the nitrocefin hydrolysis inhibition assay as well; in the presence of T405, <10% nitrocefin was hydrolyzed by BlaC compared to control or penicillin and cephalosporin subclasses (Fig. 2). T405 exhibited high affinity to PbpA, PbpB, PonA1, PonA2, DacB, DacB1, and DacB2, as the FIC_50_ of T405 against these proteins was among the lowest compared to all β-lactams (Table 2). Although T405 and faropenem belong to the penem subclass, their affinities to M. tuberculosis PBPs are distinct. For instance, the low FIC_50_ of T405 compared to faropenem against PbpA and PbpB indicates higher affinity of T405 for these two PBPs. Similarly, the FIC_50_ of T405 is also significantly lower for DacB and DacB1. On the other hand, an FIC_50_ of >4,000 of T405 against Rv1730 indicates that this putative PBP has very low affinity for T405.

## DISCUSSION

In general, the MICs of penicillins and cepholosporins against M. tuberculosis are higher, and therefore, they are not as active as carbapenems (24, 46, 47). Initial studies demonstrating poor efficacy against M. tuberculosis, when only penicillins and cephalosporins were available, became a clinical conundrum, as β-lactams exhibited potent activities against a wide spectrum of bacteria (48–50). In a seminal study to determine the efficacy of β-lactams against M. tuberculosis, Chambers et al. concluded that “β-lactamase activity” was the major factor in limiting their potency (46). Subsequent investigations led to identification of BlaC, which was proposed to encode the dominant β-lactamase (37, 41, 47, 51). Based on these studies, the lack of activity of several β-lactams against M. tuberculosis was attributed to their inactivation by BlaC. In agreement with the historical paradigm that described the relationship between β-lactams, PBPs, and β-lactamases in bacteria, BlaC was considered to be the primary source of β-lactamase activity, and PBPs were not expected to contribute β-lactamase activity.

Our findings demonstrate that interactions of M. tuberculosis PBPs and BlaC with β-lactams are not as simplistic as commonly regarded. Some β-lactams were more effectively inactivated by PBPs than by BlaC. In the first experiment, inactivation of β-lactams by PBPs was determined. In this assay, PonA1 and PonA2 hydrolyzed penicillins and carbapenems more effectively than BlaC. In the follow-up study, we used a different approach, one based on nitrocefin as a reporter of β-lactamase activity. This experiment corroborated the findings from the first experiment. These observations revealed limitations of the existing paradigm by providing evidence that proteins other than BlaC also inactivate several β-lactams effectively.

The nitrocefin hydrolysis inhibition assay demonstrated that DacB, DacB1, DacB2, and Rv2864c are relatively resistant to all β-lactam subclasses compared to other PBPs. This is a surprising finding and suggests that agents that are effective at inhibiting these proteins may further potentiate activities of β-lactams and exhibit synergy in killing M. tuberculosis. As these proteins do not belong to the classical PBPs with a transpeptidase and/or transglycosylase activity, they are not generally considered to be relevant to overall activity of β-lactams. Our data which demonstrate comparable or stronger β-lactamase activities of these four proteins (Fig. 1 and 2) and lower affinities to several β-lactams, especially penicillins (Table 1), suggest that these proteins are relevant to determining the activities of β-lactams against M. tuberculosis.

Our data also provide previously unavailable quantitative descriptions of the binding and hydrolysis of each β-lactam by M. tuberculosis PBPs, as well as a mechanistic basis for observations that are considered conundrums. The high β-lactamase activity of some PBPs is surprising and may provide at least a partial explanation to observations made by Wivagg et al. that loss of PonA1 or PonA2 or Rv2864c increases the sensitivity of M. tuberculosis to meropenem (52). Additionally, Filippova et al. reported increased susceptibility of M. tuberculosis lacking PonA1 to carbenicillin and meropenem (31). Our findings that PonA1 can hydrolyze penicillins and carbapenems at a high rate provide a basis for this observation. Flores et al. reported “hypersensitivity” of M. tuberculosis lacking PonA2 to select β-lactams (29). Since PonA2 exhibits strong β-lactamase activity against several β-lactams, our data also provide a basis for this observation. However, our data do not explain the observation of an absence of detectable β-lactamase activity in a recombinant M. tuberculosis lacking *blaC* (41). Whether PBPs with strong β-lactamase activities are expressed in abundance similar to BlaC or have a reduced affinity for β-lactams compared to peptidoglycan substrates, which would explain undetectable β-lactamase activity in M. tuberculosis lacking *blaC*, will require additional study.

Synergism between β-lactams in treating tuberculosis has been reported before and was considered unexpected (53–55). Data from our study also provide a basis for these observations. Our data demonstrate that inactivation of all PBPs by any single β-lactam does not occur and supports the hypothesis that a combination of β-lactams that can effectively inhibit multiple targets essential to M. tuberculosis viability is likely to exhibit synergism (24). Synergism between β-lactams has been reported against not only another mycobacterium, Mycobacterium abscessus (56–59), but also against Gram-positive (60) and Gram-negative organisms (61). Therefore, the precedent in other microbes and findings reported in this study provide a further basis to study whether β-lactam combinations may exhibit synergy against M. tuberculosis.

We expect our findings to stimulate reevaluation of the existing paradigm of β-lactams and M. tuberculosis. It is generally considered that β-lactamase inhibitors potentiate activities of β-lactams against M. tuberculosis primarily by inactivating BlaC (47, 62). Whether these agents also inhibit β-lactamase activities of PBPs and subsequently account for potentiation of β-lactams is not known. Our findings bring into question the presumption that β-lactamase inhibitors target BlaC only.

There are limitations to our study. Our experiments were based on *in vitro* conditions and, therefore, cannot accurately predict the ultimate potency of a β-lactam against M. tuberculosis *in vivo*, as the actual stoichiometries of PBPs and BlaC present during an infection are necessary for this determination. However, from the data on hydrolysis of each β-lactam by each PBP and the nitrocefin hydrolysis assay, the following pattern emerges: carbapenems and faropenem are least inactivated by PbpA, PbpB, DacB, DacB1, and DacB2 (highlighted in Table 1). Among β-lactams, these drugs exhibit the highest potencies against M. tuberculosis, both *in vitro* and in clinical use (46, 63, 64). Also, it has been demonstrated that carbapenems and penems are the most effective inhibitors of l,d-transpeptidases (21–24), the other enzyme class that complements activities of PBPs (15, 16, 65). On the basis of these findings, we propose the following hypothesis: in comparison to other β-lactams, the carbapenems and faropenem derive their superior potencies by more effectively inhibiting PbpA, PbpB, DacB, DacB1, and DacB2, in addition to inhibiting the l,d-transpeptidases (19–27). Another potential limitation of our study is whether the PBPs we have included in the study are comprehensive. Recent advances in β-lactam probes have identified additional proteins that are expressed in lower abundance in M. tuberculosis compared to the known PBPs (66, 67). Whether they exhibit higher catalytic activities, and therefore would play significant roles in the ultimate activity of β-lactams, will require additional study.

## MATERIALS AND METHODS

### Bacterial strains, growth media, and antibiotics.

E. coli DH5α was used for cloning, and E. coli BL21(DE3) was used for protein overexpression. Luria-Bertani broth, Tris-Cl, sodium chloride salts, and imidazole salts were purchased from Sigma-Aldrich. Powder forms of all β-lactams (Table 1) were procured from Sigma-Aldrich. Penem T405 was synthesized as described (42).

### Cloning, expression, and purification of proteins.

Genes encoding PbpA(Rv0016c), PbpB(Rv2163c), PonA1(Rv0050), PonA2(Rv3682), DacB(Rv3627c), DacB1(Rv3330), DacB2(Rv2911), Rv1367c, Rv1730c, Rv2864c, and BlaC(Rv2068c) were PCR amplified using genomic DNA (gDNA) of M. tuberculosis H37Rv with Phusion high-fidelity DNA polymerase (NEBlabs, M0530s). The amino acid sequence of each protein was analyzed using TMHMM (68) and TMPred (69) to identify putative transmembrane anchor regions. Only the gene fragments corresponding to the predicted soluble region of each protein excluding the predicted membrane domains were amplified using specific primers (Table S4) and cloned into pET28a+TEV to enable an N-terminal His6-tagged protein cleavable by tobacco etch virus (TEV) protease (19). All resulting plasmids were sequence verified and transformed into E. coli BL21(DE3) to generate expression clones. Each protein was overexpressed by inducing BL21(DE3) culture in LB broth with 0.25 mM IPTG (isopropyl-β-d-thiogalactopyranoside) overnight at 16°C with constant shaking at 150 rpm (25). The overproduced soluble proteins with His_6_ tag were purified by Ni-nitrilotriacetic acid (NTA)-based affinity chromatography with minor protein-specific optimizations as necessary (Fig. S4) (70–72). Proteins that were not soluble were treated with 1% sarkosyl at room temperature with constant low-speed shaking and followed by sonication similar to other soluble proteins. After purification, the His_6_ tag was cleaved using TEV protease. This step was followed by subjecting the protein samples to nickel affinity-column separation a second time to remove the His_6_ tag, the remaining uncleaved fusion protein, and the His_6_-tagged TEV protease (25).

10.1128/msphere.00039-22.8 FIG S4 Proteins used in the study. M. tuberculosis genes encoding PBPs and BlaC were cloned, overexpressed, and purified from E. coli. This SDS-PAGE shows the purified proteins. Download FIG S4, TIF file, 0.1 MB. Copyright © 2022 Kumar et al. 2022 Kumar et al. https://creativecommons.org/licenses/by/4.0/ This content is distributed under the terms of the Creative Commons Attribution 4.0 International license.

### β-lactam hydrolysis assay.

Each β-lactam was used at a final concentration of 1 mM, which was subsequently mixed with each PBP or BlaC each at 10 to 15 μM for a 100-μL final reaction volume in 10 mM Tris-Cl buffer, pH 7.4. The β-lactam ring opening reaction was monitored for 120 min, 25°C, using a SpectraMax 250 spectrophotometer. Each assay was performed in triplicate under identical reaction conditions. For each protein and β-lactam combination, the rate of β-lactam hydrolysis was determined, and the composite hydrolysis was computed for each β-lactam class by each PBP and BlaC (Fig. 1, Table 1, Table S1 and S2, Fig. S1). Means and standard errors are reported.

### Nitrocefin hydrolysis assay.

Nitrocefin (Calbiochem; no. 484400) is a chromogenic β-lactam whose hydrolysis is quantified by measuring the transition from the substrate form (390 nm) to the product (496 nm) (43). Nitrocefin was used at 100 μM, in 10 mM Tris-Cl, pH 7.4, as previously described (25, 41). Each protein (10 μM) was incubated with a β-lactam (200 μM) for 30 min, 25°C, prior to addition of nitrocefin in 100-μL final volume. The reaction was monitored at 496 nm for 120 min, 25°C. Kinetic parameters of the enzymes were determined by methods described earlier (25, 70).

### BOCILLIN FL fluorescence inhibition assay.

BOCILLIN FL (Thermo Fisher Scientific; no. B13233) is a fluorescent penicillin (λ_max_ = 504 nm) (44). BOCILLIN FL was used at 50 μM in Tris-Cl, pH 7.4, and incubated with a fixed amount of each protein (∼200 pmol). β-lactams were used in a gradient concentration range 0 to 4,000 μM (Table S3). Each protein was incubated with a β-lactam at 25°C for 30 min, followed by BOCILLIN FL addition. The reaction mixture was incubated for an additional 30 min, 25°C, in the dark, followed by reaction quenching with Laemmli buffer and heat denaturation at 95°C, 5 min. This mixture was electrophoresed on 14% SDS-PAGE, and BOCILLIN fluorescence was imaged using a GelDoc imager (Bio-Rad). The fluorescence intensity of BOCILLIN FL in each protein band was quantified using ImageJ. Next, the SDS-PAGE gel was stained with Coomassie brilliant blue dye to verify the presence and stability of each protein.
