# Supplementary figures and images for: Penicillin Binding Proteins and β-Lactamases of Mycobacterium tuberculosis: Reexamination of the Historical Paradigm
Source: mSphere. 2022 Feb 23;7(1):e00039-22. doi: 10.1128/msphere.00039-22 (PMC8865919; doi:10.1128/msphere.00039-22)

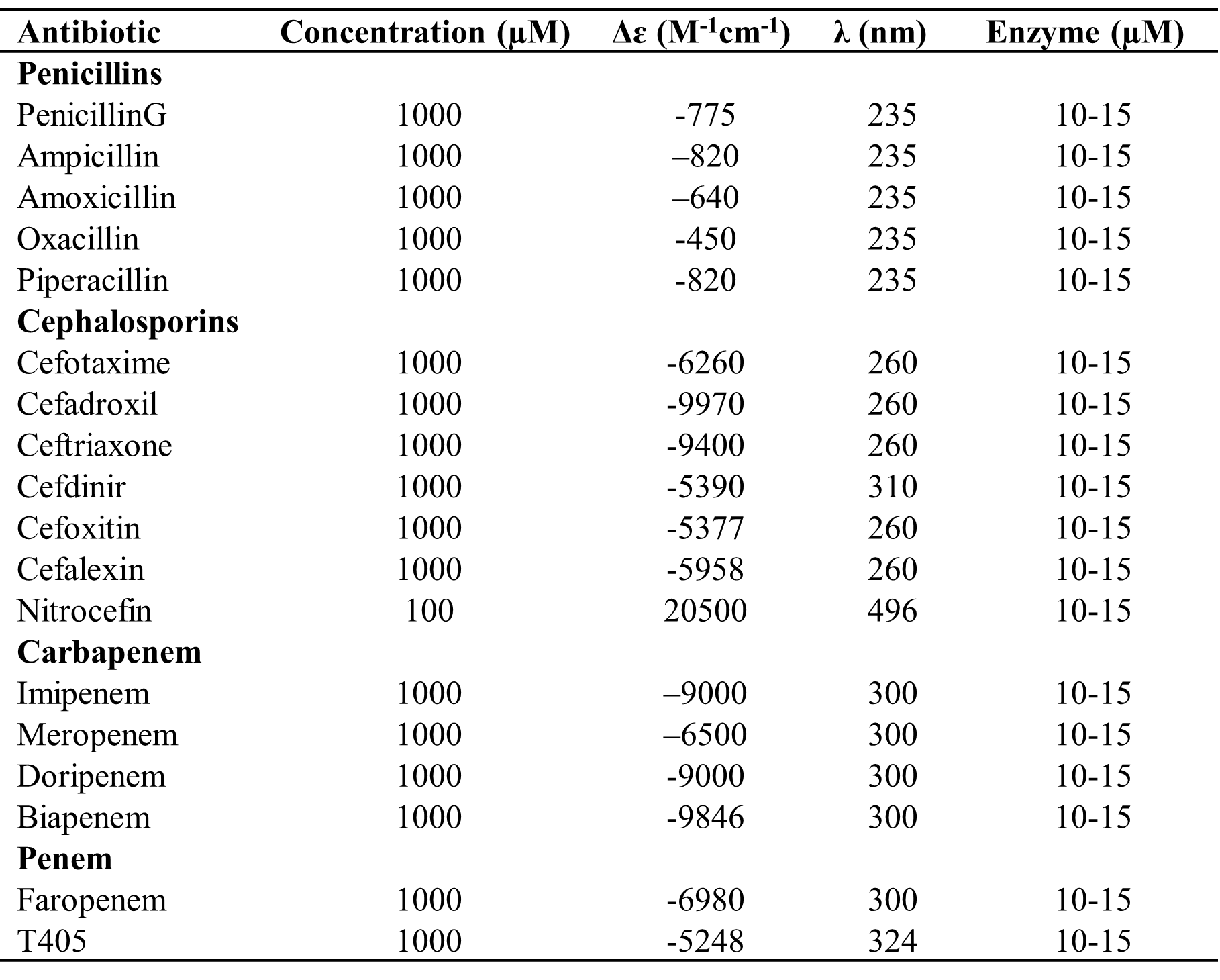

Supplement: TABLE S1 [file msphere.00039-22-st001.tif]

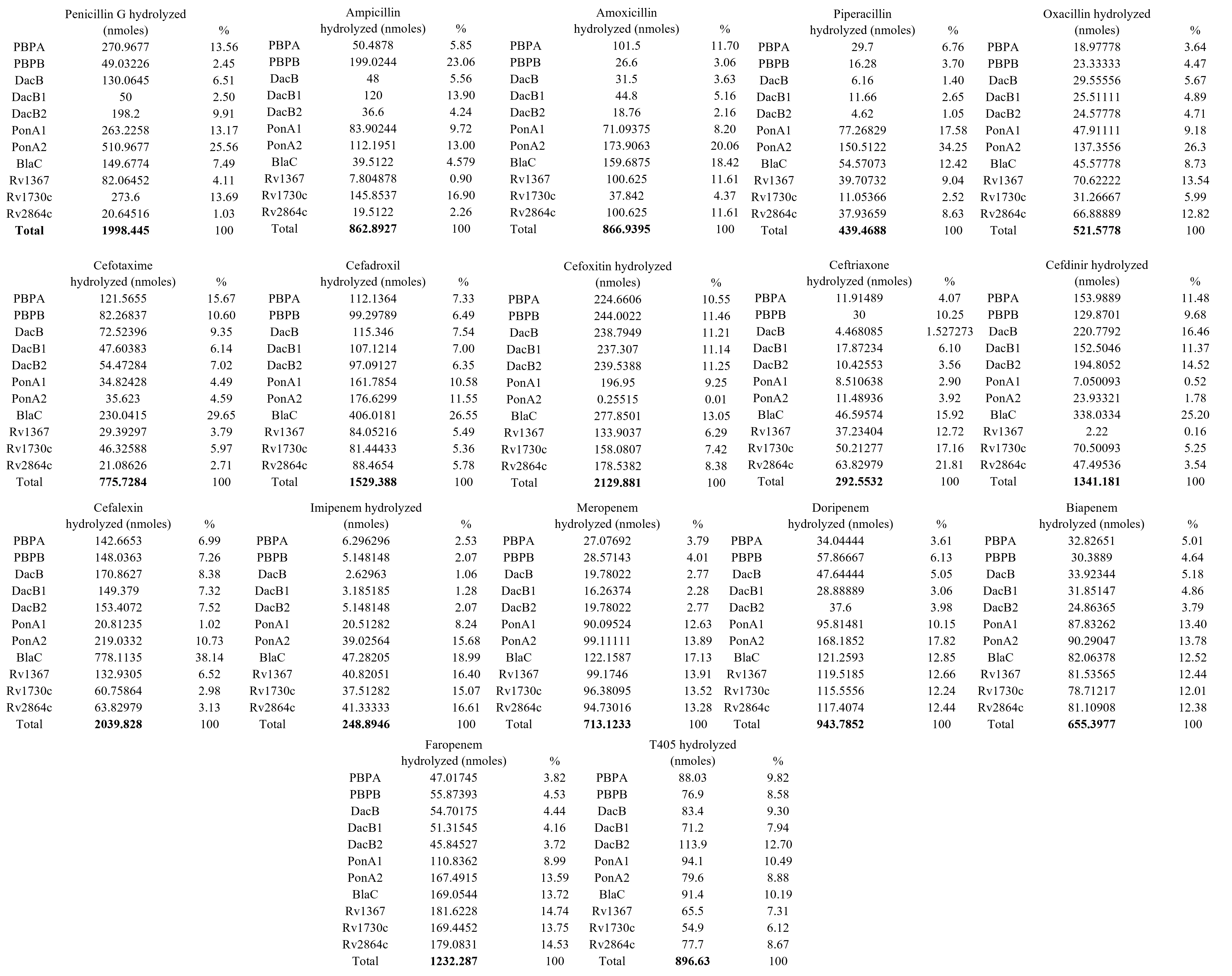

Supplement: TABLE S2 [file msphere.00039-22-st002.tif]

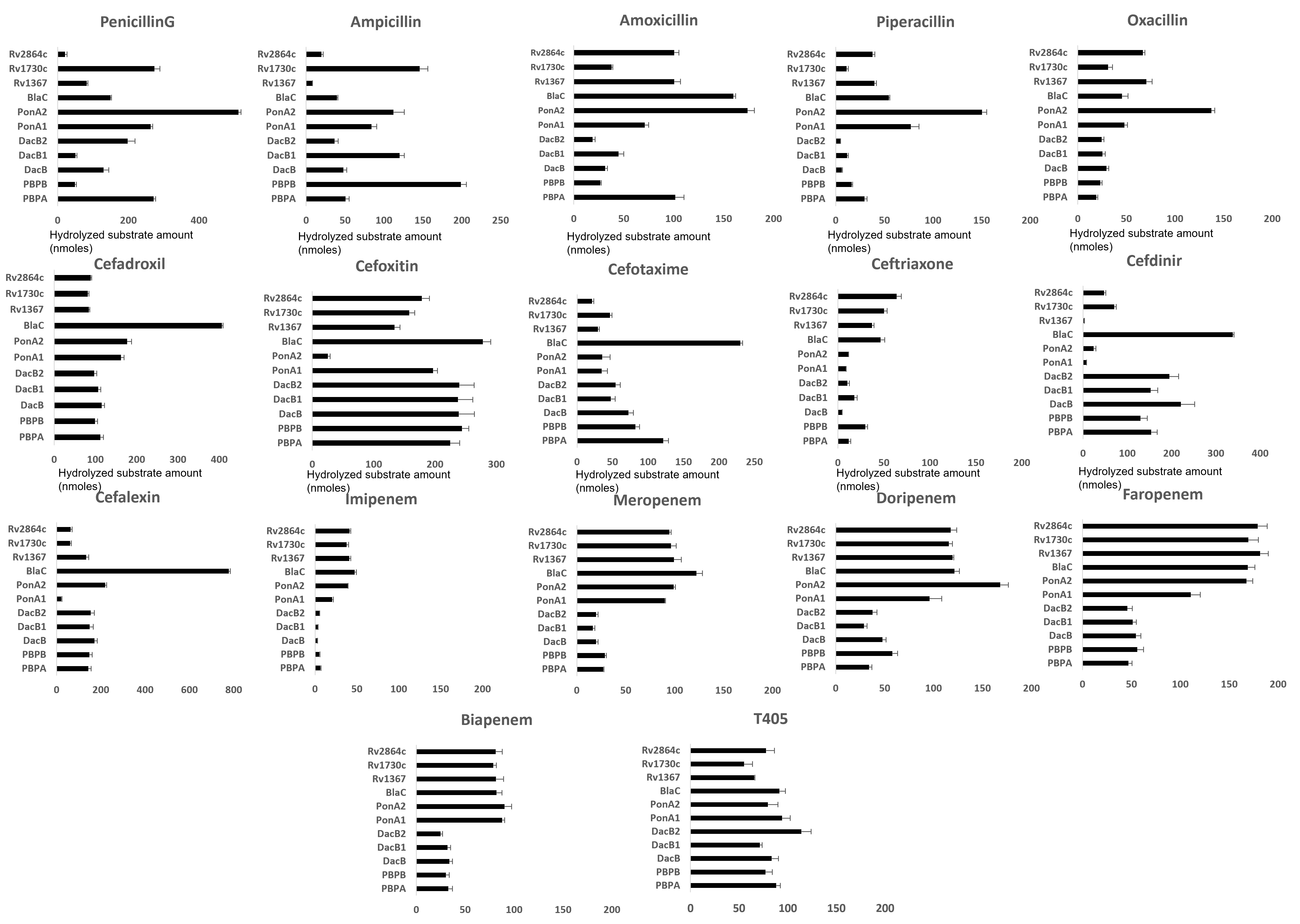

Supplement: FIG S1 [file msphere.00039-22-sf001.tif]

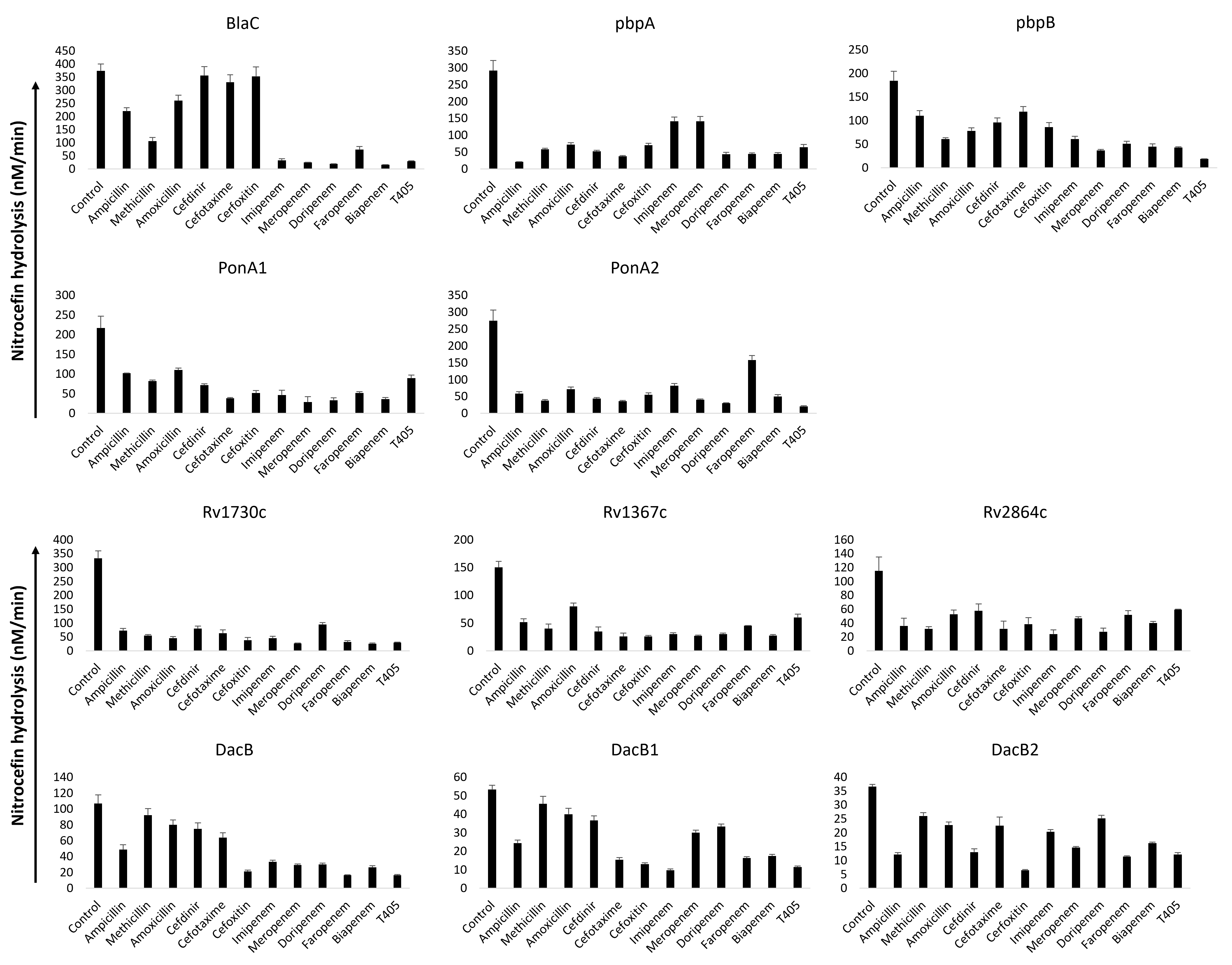

Supplement: FIG S2 [file msphere.00039-22-sf002.tif]

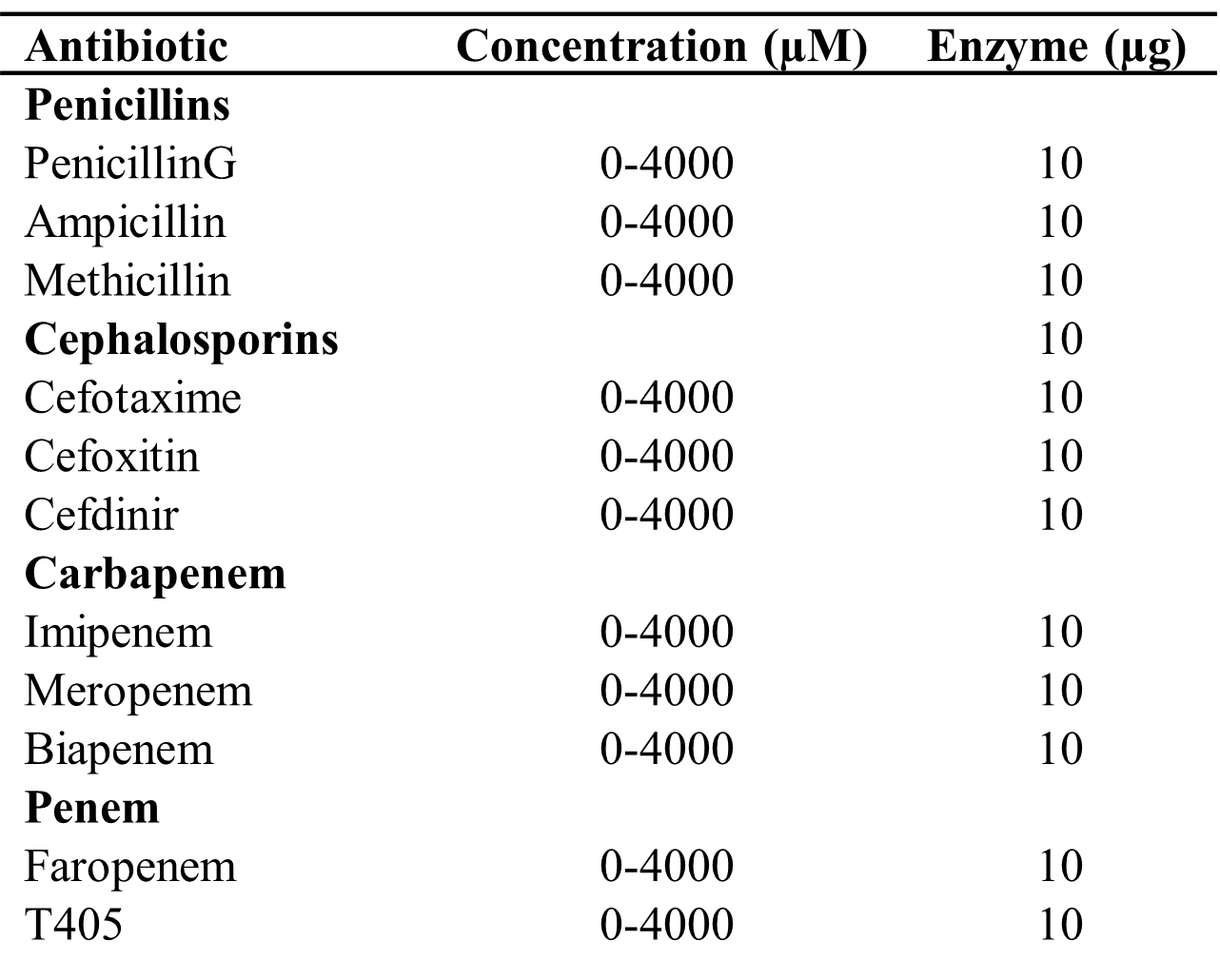

Supplement: TABLE S3 [file msphere.00039-22-st003.tif]

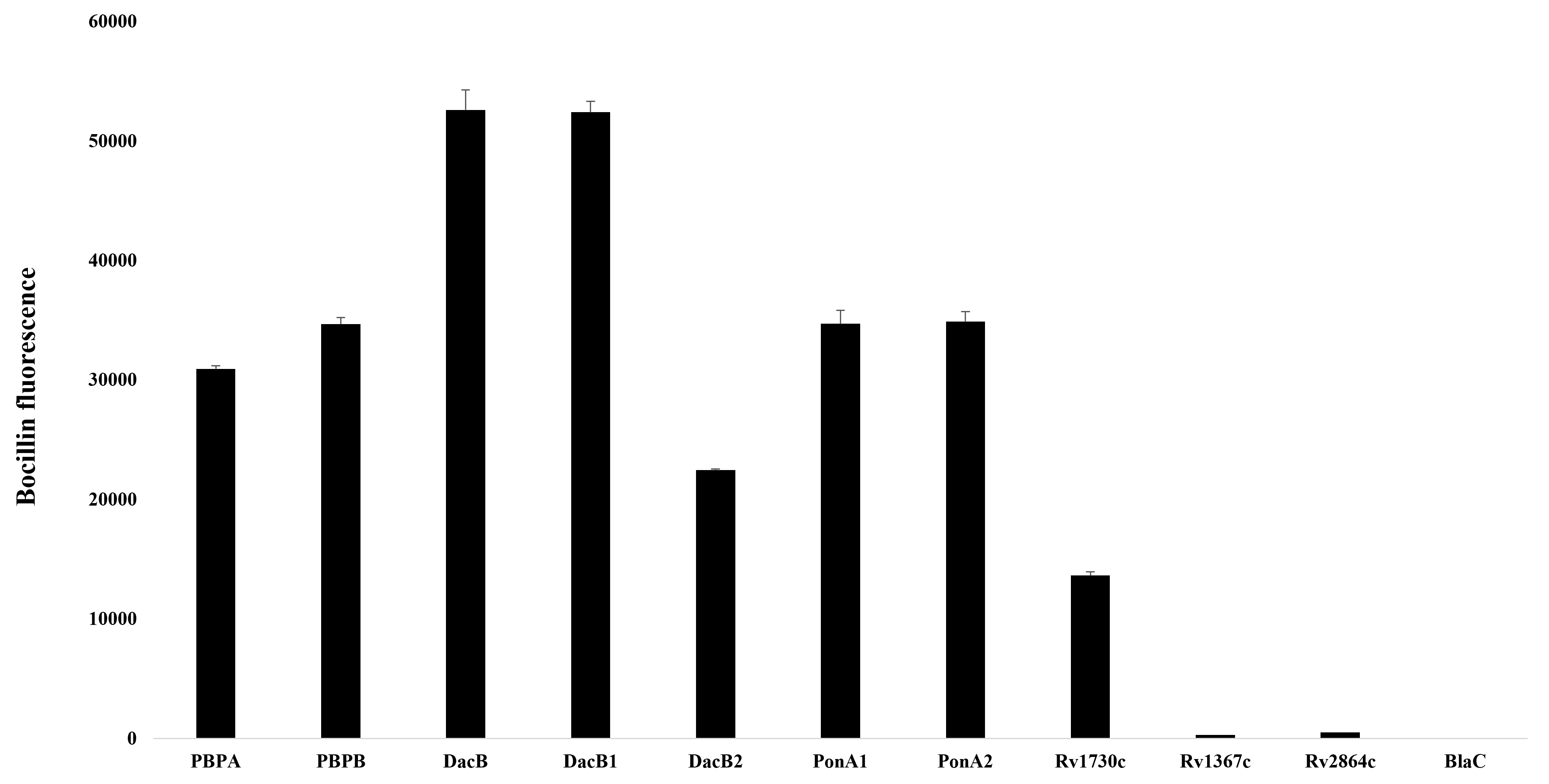

Supplement: FIG S3 [file msphere.00039-22-sf003.tif]

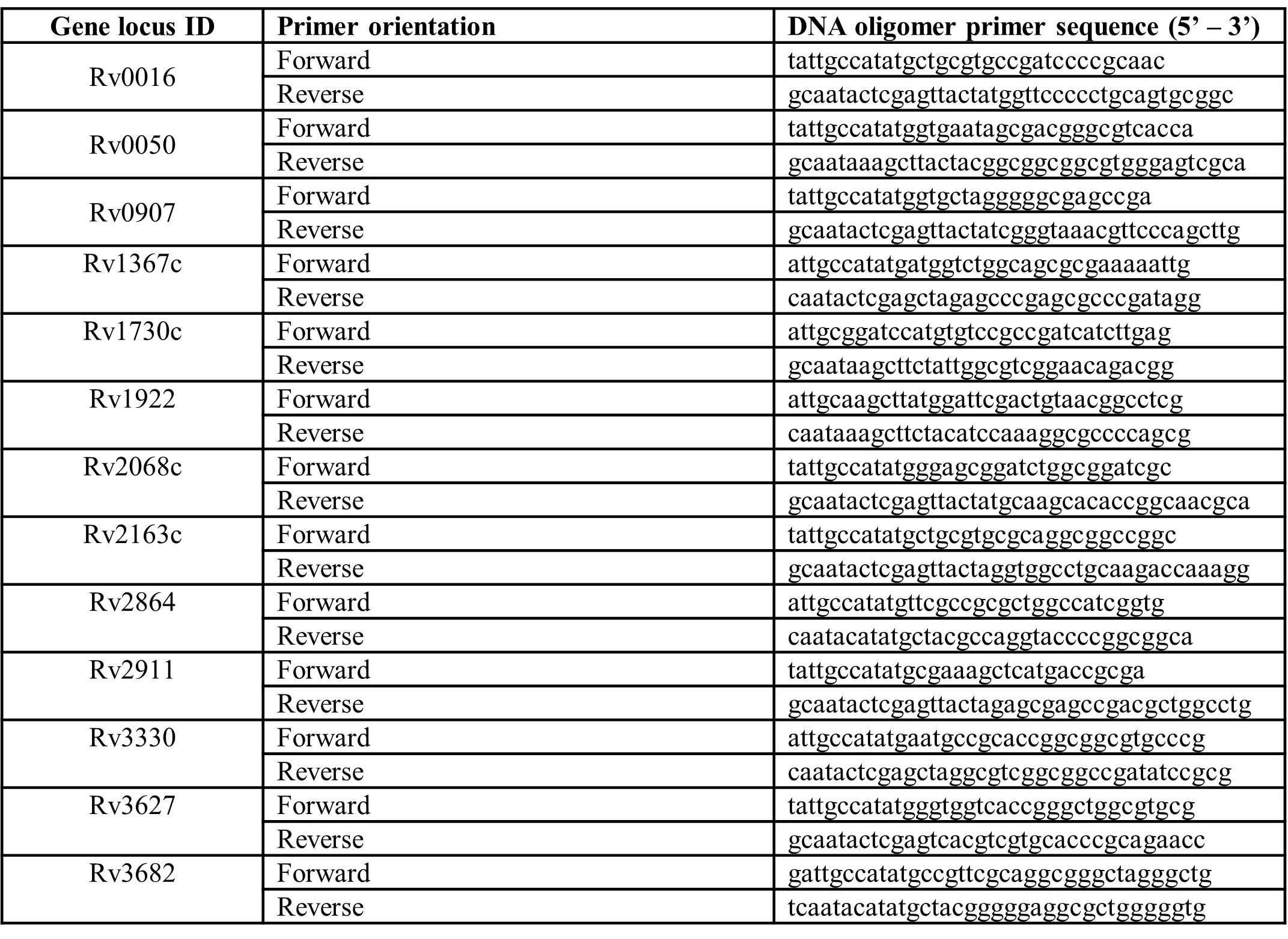

Supplement: TABLE S4 [file msphere.00039-22-st004.tif]

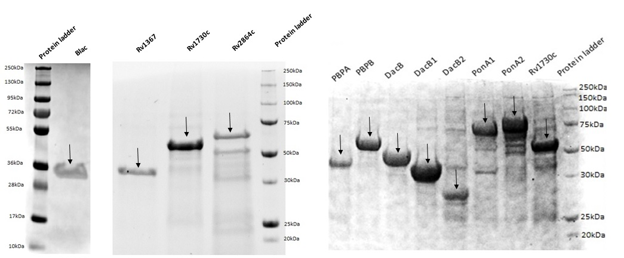

Supplement: FIG S4 [file msphere.00039-22-sf004.tif]
